# “Husa” as a Remedy for the Opium Habit

**Published:** 1898-07

**Authors:** 


### "HUSA" AS A REMEDY FOR THE OPIUM HABIT.

The February number of the *Texas Courier-Record of Medicine* opens with a remarkable account by Dr. W. W. Winthrop, of Fort Worth, of his experience with an unclassified plant which he thinks may be indigenous to the everglades of Florida, one known by the name of "husa." He first observed its use in connection with that of the arrow-leaved violet, *Viola hastata*, a plant that is found growing from Canada to Florida and westward to Arkansas.

Dr. Winthrop says that when he was living in Florida he often saw strange things done with snakes. He mentions a negro who was visiting the towns on the Indian River, giving exhibitions with two immense, terrible-looking rattlesnakes. He would irritate the snakes and allow them to bite him in any part of his person indicated by anybody who was willing to pay twenty-five cents to "see the show." Dr. Winthrop declares that the snakes had their fangs, which must have been "about three inches long." Furthermore, the negro would allow himself to be bitten by any snake that a person might catch and bring to him. Dr. Winthrop saw him bitten several times by moccasins that had just been caught. After each bite the negro would take a mouthful of some herbs which he carried in a little bag, maintaining that the herbs counteracted the venom. "He had none of the herbs for sale, the 'whole show' consisting of seeing the snakes bite." By the judicious use of money and whisky Dr. Winthrop succeeded in worming out of the negro this information: "Boss, de is viellies an' huser, an' I gets 'em from de Semmes in de dales."

"Semmes in de dales" evidently meant the Seminole Indians of the everglades, and to them the author betook himself for information. "But," he says, "though I used every means I could think of to get information about their remedies for snake-bite, I could elicit nothing from the Indians, men or women; neither the young men and boys, with whom I hunted day and night, could I induce to speak, though every one of them knew what I wanted. I was a stranger to them and that was enough. These Indians will not give a stranger information about anything; they must know who and what he is first." Just as he was about to give up disheartened, the author fell in with a Scotch physician who was botanizing in the everglades. This gentleman interpreted "viellies" as meaning "the spear-eared violet, *Viola sagitata*," and "huser" as denoting an unclassified plant variously called "husa," "hoosa," "yousa," and "yusee" in Florida.

Dr. Winthrop states that *Viola sagitata* has long been known as possessing properties antidotal to snake poison. He gives the following account of "husa": "It is an unclassified plant of a dirty whitish-green color, about two or three inches long. It has at its summit a ball-like white formation. Where the flower should be, this is hard, slightly lobulated, and is to all appearances like a small cauliflower. It grows in clumps in moist, shady places, particularly on the hammocks at the roots of the cabbage palms. It is of a low order of plants, above the mosses; it is, I believe, a cryptogam. It is possibly indigenous to the everglades, for I hunted for it in vain in many large hammocks in Florida. From Dr. McGregor I learned that it is a perfect antidote for all snake-bites, stings of insects, etc.; also an antidote for narcotic poisons. It is the most diffusible stimulant known, acting immediately."

Dr. Winthrop states that he has subjected the plant to various tests, and has found it an infallible cure for the opium habit. He says: "It takes the place of opium or morphine. Supporting the patient fully, it is sedative but not narcotic. It produces slight elation, but no somnolent effect. To use the illustration of one physician who cured himself of the opium habit with it, a habit of twenty-three years' standing," one who was using forty grains of morphine sulphate daily, "'It makes a man feel just as easy and comfortable as one feels after a satisfying meal.' As soon as I learned its properties I sent some of the husa plant to several doctors I knew who used morphine; they one and all pronounced it 'a perfect success.' I have never known of a failure when the patient wanted to be cured. In the hand of a careful physician, this remedy will be found efficient in the worst cases of drug addiction."

It is to be hoped that the botanists will give us some information about "husa," and that its medicinal virtues may be inquired into systematically.—*New York Medical Journal.*

